# Interactive Brain Activity: Review and Progress on EEG-Based Hyperscanning in Social Interactions

**DOI:** 10.3389/fpsyg.2018.01862

**Published:** 2018-10-08

**Authors:** Difei Liu, Shen Liu, Xiaoming Liu, Chong Zhang, Aosika Li, Chenggong Jin, Yijun Chen, Hangwei Wang, Xiaochu Zhang

**Affiliations:** ^1^School of Humanities and Social Science, University of Science and Technology of China, Hefei, China; ^2^Department of Education, Hefei University, Hefei, China; ^3^School of Foreign Languages, Anhui Jianzhu University, Hefei, China; ^4^Department of Mechanical and Automation Engineering, The Chinese University of Hong Kong, Hong Kong, China; ^5^Department of Social and Behavioural Sciences, City University of Hong Kong, Hong Kong, China; ^6^CAS Key Laboratory of Brain Function and Disease, and School of Life Sciences, University of Science and Technology of China, Hefei, China; ^7^School of Chemistry and Materials Science, University of Science and Technology of China, Hefei, China; ^8^Hefei Medical Research Center on Alcohol Addiction, Anhui Mental Health Center, Hefei, China; ^9^Academy of Psychology and Behavior, Tianjin Normal University, Tianjin, China

**Keywords:** social interaction, EEG-based hyperscanning, inter-brain synchrony, phase coherence, inter-brain activities

## Abstract

When individuals interact with others, perceived information is transmitted among their brains. The EEG-based hyperscanning technique, which provides an approach to explore dynamic brain activities between two or more interactive individuals and their underlying neural mechanisms, has been applied to study different aspects of social interactions since 2010. Recently there has been an increase in research on EEG-based hyperscanning of social interactions. This paper summarizes the application of EEG-based hyperscanning on the dynamic brain activities during social interactions according to the experimental designs and contents, discusses the possibility of applying inter-brain synchrony to social communication systems and analyzes the contributions and the limitations of these investigations. Furthermore, this paper sheds light on some new challenges to future EEG-based hyperscanning studies and the emerging field of EEG-based hyperscanning for pursuing the broader research field of social interactions.

## Introduction

Social interaction, a fundamental part of our daily life, is at the core of human behaviors (Dumas, 2011; Dumas et al., 2014). There are many social interactions in our daily lives, which involves different kinds of interpersonal synchronies. For example, we synchronize our footsteps with those of our partners unconsciously when we walk together (Reddish et al., 2013). This phenomenon is considered as interpersonal synchrony. Synchronous behaviors, playing a central role in establishing and promoting social ties, are socially important. In addition, the degree of synchrony predicts subsequent affiliation ratings (Reindl et al., 2018). Some researchers found that there was a close relationship between neural dynamics and interpersonal behavioral synchronization (Hove and Risen, 2009). Based on those findings, some researchers are dedicated to exploring the mechanism of interpersonal synchrony and the functional significance of inter-brain synchrony in interpersonal interactions.

Through social interactions with others, human beings know each other and form a family or a state (Decety and Lamm, 2007). Although the social nature of human beings was noticed thousands of years ago, the studying of brain activities during social interactions in neuroscience has only been carried out for about 10 years (Hari and Kujala, 2009). In recent years, social neuroscientists suggest that the dynamic brain activities between two or more interactive individuals should be analyzed in order to provide a window into how their minds (Hari et al., 2015). A technique called hyperscanning or pseudo-hyperscanning is used to assess the level of between-brain coupling, which requires the measurement of brain activities of two or more participants involved in social interactions. Hyperscanning is a measurement of brain activities of participants at the same time, and pseudo-hyperscanning is a similar measurement but measures each participant at a time (Schoot et al., 2016). The first study on dynamic brain activities between individuals by virtue of electroencephalography (EEG) can be traced back to an experiment conducted by Duane and Behrendt (1965). It took the lead in using the EEG to record twins' brain activities simultaneously and calculate the correlation between EEG traces. From then on, several researchers investigated the dynamic brain activities and reported correlated brain signals by using simultaneous electroencephalographic recordings from interactive participants. However, these studies on how two brains interact with each other used offline designs and the subjects were isolated from one another without actually taking part in social interactions due to technological limitations (Kohler, 1969; Perez-Rincon et al., 1981).

The idea of recording multi-subjects' brain activities simultaneously was proposed by Montague et al. (2002) and it was called the “hyperscanning” technique. The term “hyperscanning” refers to simultaneous recording of hemodynamic or neuro-electric activity of the brains from multiple subjects involved in social interactions. By means of cognitive neuroscience equipment (e.g., EEG, fMRI, fNIRS), the “hyperscanning technique” has the potential to explore interpersonal brain mechanisms underlying neuronal correlation between interaction during two or more people taking part in social interactions (Balconi and Molteni, 2015). Combined with fNIRS, fMRI and EEG, the study on the neural mechanism of interpersonal social interactions has currently gained momentum in the young field of social neuroscience since 2002 (Konvalinka and Roepstorff, 2012; Babiloni and Astolfi, 2014; Koike et al., 2015; Schoot et al., 2016; Xue et al., 2018). Conventionally, the fMRI- or fNIRS-based hyperscanning has the general drawback of the low temporal resolution due to the inertia of the bold response, whereas EEG has finer temporal resolution and thus becomes the most frequently used technique in hyperscanning studies (Koike et al., 2015). One of the advantages of EEG is that it has finer temporal resolution than fMRI. EEG provides the opportunity to record activation on the millisecond scale (Spiegelhalder et al., 2014). Another advantage of EEG is it allows us to observe the inter-brain neural synchronization in more natural settings despite the argument that EEG is susceptible to head movements (Koike et al., 2015). Therefore, studies of EEG-based hyperscanning on social interactions have been extended increasingly, from simple imitative interactions to complicated affective communications in the last decade.

Thus, this review firstly introduced the social interaction and its importance as well as provided an overview of the application of EEG-based hyperscanning technique on the dynamic brain activities during social interactions. Then, this review introduced four specific domains of inter-brain activities according to the experimental designs and contents. Next, this review analyzed the contributions as well as the limitations of these investigations and shed light on the new challenges to future EEG-based hyperscanning studies. Furthermore, this review discussed the upcoming field of EEG-based hyperscanning to pursue a broader study field of social interactions.

## Inter-brain activities of joint action

In recent years, with the development of EEG-based hyperscanning technique, several independent research teams have used movement synchronized task (Tognoli et al., 2007; Lindenberger et al., 2009; Dumas et al., 2011; Yun et al., 2012), leader-follow task (Sänger et al., 2012; Konvalinka et al., 2014), speech rhythm synchronization task (Kawasaki et al., 2013), cooperative task (Balconi et al., 2015) and actor-observer interaction paradigm (Ménoret et al., 2014) to explore the neural mechanisms of social coordination. These are all related to joint actions and believed to involve a variety of mechanisms (Della Gatta et al., 2017). In order to know how brain-to-brain interacted with each other during joint actions, some researchers investigated the dynamic brain activities between pairs of subjects while executing spontaneous imitation movements toward the vision of each other's actions (Dumas et al., 2010). The results showed that the alpha–mu band showed the strongest inter-brain synchrony among the right centroparietal regions. Another unconsciously synchronized fingertip movement experiment was conducted to explore the mechanisms of body movement synchrony (Yun et al., 2012).

In order to reduce similarities in movement and perception which may enhance inter-brain synchrony of the experiment, Sänger et al. (2012) used a modified leader-follower task with two guitarists playing in two voices. The enhanced phase locking, within- and between-brain phase coherence was found during musical coordination periods, especially at frontal and central sites. The results extended previous findings and attributed between-brain phase coherence to interpersonal action coordination rather than interpersonal similar action. However, some EEG-based hyperscanning studies showed that asymmetric brain-coupling^1^ patterns of leader-follower participants in a dyad during coordinated movements (Dumas et al., 2010). The asymmetric phenomena were also emerged from some studies of decision-making in game contexts (Balconi and Vanutelli, 2016). This asymmetric pattern of coupling may be explained by the differential roles of the partners during the interaction, and the participants may have different expectations for the assigned roles. Whether the asymmetric brain-coupling pattern is the mechanism of leader-follower communication remains unresolved, which can be explored in future researches.

In addition, the inter-brain synchrony was also found in other experiments of interpersonal behavioral coordination. For example, in a cooperative and competitive task, the inter-brain synchrony between subjects was significantly higher when they cooperated with each other than that when they were in the competitive condition (Davis et al., 2016). Kuhlen et al. (2012) revealed the coordination of brain activities between the speakers and listeners during verbal communications. Kawasaki et al. (2013) investigated the relationship of brain rhythm synchronization during speech rhythm synchronization between individuals and found the inter-brain synchrony of theta/alpha (6–12 Hz) amplitudes in the temporal and lateral-parietal regions in each pair. Moreover, Ménoret et al. (2014) found that the suppression of beta oscillations was observed in the actor's EEG and the observer's EEG rapidly after the onset of the actor's movement during a face-to-face actor-observer interaction paradigm, and this suppression was stronger for the observer in the interactive than in the non-interactive context independent of the act conducted by a human or a robot.

Based on quantifying functional similarities or temporal synchronization between brains during social interactions, most results attributed inter-brain synchrony or phase coherence to interpersonal action coordination.

## Inter-brain activities of shared attention

Among social signals, the non-verbal signals are deemed to be crucial visual cues for communicative intentions (Jahng et al., 2017). During these processes, people share the same perspective with one another, and this phenomenon is called shared attention (Shteynberg, 2018).

Mutual gaze and shared attention play an essential role in our abilities to detect others' focuses of interest, as well as to infer their intentions, desires and thoughts. The importance of mutual gaze and shared attention on the development of social cognition has been underlined (Koike et al., 2016). To investigate the neural mechanisms of interpersonal shared attention, researchers measured the brain activities of two people who engaged in actual mutual gaze or shared attention experimental task with inter-subjective sharing reciprocal information without words by recording simultaneously dual-EEG. Lachat et al. (2012) set up a live shared attention paradigm to investigate the influence of shared attention on oscillatory activities within the alpha-mu (8–12 Hz) frequency band. Compared with the no-shared attention periods, a decrease of 11–13 Hz signal was found during the shared attention periods over a large set of left centroparietal electrodes extending to occipital electrodes. Another EEG-based hyperscanning study was performed by Leong et al. (2017) to verify whether direct gaze increased neural coupling between adult-infant partners during social interactions. Dikker et al. (2017) found that the highest pairwise alpha coherence emerged in student pairings who sat face-to-face compared to the other two student pairings (adjacent and no face-to-face or no adjacent) and the inter-brain synchrony between students consistently predicted class engagement and social dynamics.

The studies mentioned above supported the view that alpha frequency band was involved in visual processing (van den Heuvel et al., 2018), arousal and attentional mechanisms (Foxe and Snyder, 2011). People exchange reciprocal information via eye-to-eye contact and act according to the interpretation of the information. The results in certain degree showed that eye contact enhanced neural coupling between interactive individuals during social interactions. The conclusion was verified by the experiment about autism spectrum disorders (Yates and Couteur, 2016).

## Inter-brain activities of interactive decision-making

Interactive decision-making is defined as the dynamic process of making choices depending on the antecedent decision behaviors of the partner and other social cues in interactive tasks. It is one of the most omnipresent activities in human beings (Nummenmaa et al., 2018). The decision-making process always requires higher degree of cognitive involvement between interactive individuals in real life. Such an activity involves goal-directed behaviors, social cognition, and theory-of-mind abilities (Gilam and Hendler, 2016). By using EEG-based hyperscanning, a series of studies in game contexts provide abundant evidences for the neural process of interactive decision-making during social interactions.

For example, Balconi and Vanutelli (2016) for the first time explored the neural process during interactive decision-making with EEG-based hyperscanning technique. The experiment was performed in five groups of four subjects during a cooperative card game that involved groups of two subjects against other two. The game was played with two teams of subjects sitting at north and south against those two sitting at east and west. The results showed that causal links emerged from prefrontal areas of the different subjects when they were performing cooperative games in different frequency bands. One of the remarkable things among the Prisoner's Dilemma experiments is the controversial result of the connectivity between the two brains in the defect condition during interactive decision making. The inter-brain synchrony refers to dynamical similarity in brain signals. Even competitive behavior could lead to them if the people need to represent the same information at the same moment. Jahng et al. (2017) found that the pattern of inter-brain connectivity in the cooperation condition was denser than in the defect condition when the individuals engaged in the Prisoner's Dilemma game. Cooperation and defection are different types of social interactions. Some studies also found the inter-brain links with EEG-based hyperscanning during interactive decision-making (Hu et al., 2018). The result also emerged in previous fMRI-based hyperscanning studies in interactive decision-making (King-Casas et al., 2005). For example, Hu et al. (2018) stepped further to compare inter-brain synchrony between H-H (human played the Prisoner's Dilemma game with partner) with H-M (human played the Prisoner's Dilemma game with computer) and found that there was a higher rate of cooperation and larger theta/alpha-band inter-brain synchrony in H-H condition. These findings were in keeping with some neuroimaging studies which suggested cooperation promotes inter-brain synchrony (Pan et al., 2016).

In this part, we discussed how two brains interacted with each other when individuals engage in a more complicated social activity—interactive decision-making. There are two hypotheses proposed to explain the emergence of inter-brain synchrony in interactive decision making: the cooperative interaction hypothesis and the similar task hypothesis (Hu et al., 2018). A line of evidence has demonstrated that neural activities of two individuals are more synchronized when they perform cooperative interactions (Tognoli et al., 2007; Lindenberger et al., 2009; Dumas et al., 2011; Yun et al., 2012).

## Inter-brain activities of affective communication

Affective communication is a complex process during which interactive individuals express and perceive emotional signals and exchange information about internal affective states (Symons et al., 2016). It is a form of emotional support having direct and indirect effects on the stress process (Viswesvaran et al., 1999). Emotions play an important role in regulating and motivating a person's thoughts, feelings, and behavior in almost every aspect of people's life. Interpersonal emotions are evoked when we implicitly or explicitly reflect on ourselves and evaluate ourselves in the context of our surrounding social world (Symons et al., 2016). Müller and Lindenberger (2011) found oscillatory couplings of cardiac and respiratory activities among singers and conductor while they were engaging in choir singing. It seems that favorable affective communication between two individuals is closely related to their physiological states. However, the interactive mechanism of the two dynamic brains is unclear as to affective communication.

Physicians' affective communication has a supportive function particularly effective in situations where individuals lack control (Ommen et al., 2008; Dumas et al., 2012; Abrams et al., 2013; Fowler et al., 2013; Novembre et al., 2017). By means of the EEG-based hyperscanning technique, Müller and Lindenberger (2011) found that theta-alpha hyper-brain networks bound the two brains of kissing partners together with a method of network construction based on the cross-frequency coupling. Thus, it can be inferred that brain-to-brain coupling is a neural marker for interpersonal communication of affection. It is noticeable that there is a relatively weaker inter-brain coupling between the right parietal regions of the female partner and the right parieto-occipito-temporal areas of the male partner in the control (no-touch-no-pain) condition. The inter-brain coupling pattern is in line with the previous findings of interpersonal action coordination (Dumas et al., 2010; Konvalinka et al., 2014). Wang et al. (2015) proposed that co-presence of two speakers could result in their autonomic physiological coupling.

Human emotional experience naturally occurs while interacting in a spontaneous, dynamic and response-contingent fashion with other humans (Gilam and Hendler, 2016). Based on above-mentioned, it can be assumed that the inter-brain coupling pattern in the control condition may constitute a basic interpersonal interaction. However, there are few tasks related to interactive affection communication, which needs future exploration.

## Future challenges and directions

Many studies with EEG-based hyperscanning have elucidated that the inter-brain synchrony as a result of ongoing social interactions is more directly and precisely, from coordinated behaviors to affective communication, and have focused on describing the specific time and frequency ranges of the neural processing (Dumas et al., 2010; Kawasaki et al., 2013). Based on quantifying functional similarities or temporal synchronization between brains during social interactions, most studies regarded inter-brain synchrony or phase coherence as an important index of interpersonal interaction and attributed inter-brain synchrony or phase coherence to interpersonal action coordination (Konvalinka et al., 2014). With different experimental tasks, the findings showed that the inter-brain synchrony got across different frequencies. With the portability of EEG devices, people were able to interact naturally and the inter-brain effect was recorded in a very natural setting (Astolfi et al., 2011). The design of social interactive experiments mentioned is more realistic, and studies on social interactions have been extended to a wide range of fields. Though neuroscience has made great progress in recent decades, we only have a preliminary understanding of how two brains interact with each other during social interactions. All studies this review mentioned can be seen in Table 1. For the purpose of studying and comprehending the neural process of social communications in greater depth, we need to focus on some challenges as well as future directions.

**Table 1:** List of the studies mentioned with EEG-based hyperscanning methodologies.

**Research area**	**Authors, year, journal**	**Task instructions**	**Number of subjects**	**Results**
Joint action	Balconi et al., 2015, Brain and Cognition	Participants were required to observe affective pictures during EEG recording, and they should attend to them the entire time of exposition.	20	An increased theta activity for negative stimuli in the right more than in the left side was observed.
	Balconi and Vanutelli, 2016, frontiers in Psychology	Participants were required to develop a strategy to obtain a better outcome than a competitor (in term of error rate, and response time, RT).	24	A decreased left alpha activity (increased brain response) for post-feedback compared to pre-feedback condition was observed.
	Dumas et al., 2010, PLoS ONE	Participants were engaged in spontaneous imitation of hand movements.	18	Symmetrical increase in PLV was found between the right parietal regions of the model (CP6, P8) and of the imitator (CP6, P4, P8) in the alpha-mu frequency band. The central region (FC1, Cz) of the model's brain and the parieto-occipital brain region (P8, PO2, PO10) of the imitator were synchronized in the beta frequency band. A wide frontal central area (F4, FC2, Czar, C4, CP6) of the model's brain was synchronized with the parietal area (CP2, PZ, P4, P8, PO2, PO10) of the imitator's brain for the gamma frequency band.
	Kawasaki et al., 2013, Scientific Reports	Alternating speech tasks.	40	Speech rhythms were more likely to become synchronized in human–human tasks than human–machine tasks. Moreover, theta/alpha (6–12 Hz) amplitudes synchronized in the same temporal and lateral-parietal regions in each pair. Behavioral and inter-brain synchronizations were enhanced after human–machine tasks.
	Konvalinka et al., 2014, NeuroImage	A synchronized finger-tapping task.	18	The interactive condition was characterized by a stronger suppression of alpha and low-beta oscillations over motor and frontal areas in contrast to the non-interactive computer condition.
	Kuhlen et al., 2012, frontiers in Human Neuroscience	Speakers were given the task to make the stories interesting and fun for future listeners to listen to.	12	The EEG is more similar among listeners attending to the same speaker than among listeners attending to different speakers, indicating that listeners' EEG reflects content-specific information. Listeners' EEG activity correlates with the attended speakers' EEG, peaking at a time delay of about 12.5 s. This correlation takes place not only between homologous, but also between non-homologous brain areas in speakers and listeners.
	Lindenberger et al., 2009, BMC Neuroscience	Eight pairs of guitarists played a short melody together.	18	Phase synchronization both within and between brains increased significantly during the periods of preparatory metronome tempo setting and coordinated play onset. Phase alignment extracted from within-brain dynamics was related to behavioral play onset asynchrony between guitarists.
	Ménoret et al., 2014, Neuropsychologia	Participants were instructed to perform object-directed movements toward one of three different objects: a box, a saucer and a candle-holder.	40	For the observer, an observation related motor related potentials was measured in all conditions but was more negative in the interactive context over fronto-central electrodes. Moreover, this feature was specific to biological actions. Concurrently, the suppression of beta oscillations was observed in the actor's EEG and the observer's EEG rapidly after the onset of the actor's movement. Critically, this suppression was stronger in the interactive than in the non-interactive context despite the fact that movement kinematics did not differ in the two context conditions.
	Sänger et al., 2012, frontiers in Human Neuroscience	Participants sat face-to-face to each other. One participant was assigned the leading role, meaning that he or she was responsible for bringing the other in and determining the playing tempo. The follower was asked to exclusively orient himself toward the leader.	22	Phase locking as well as within-brain and between-brain phase-coherence connection strengths were enhanced at frontal and central electrodes during periods that put particularly high demands on musical coordination. Phase locking was modulated in relation to the experimentally assigned musical roles of leader and follower, corroborating the functional significance of synchronous oscillations in dyadic music performance. Graph theory analyses revealed within-brain and hyperbrain networks with small-worldness properties that were enhanced during musical coordination periods, and community structures encompassing electrodes from both brains (hyper-brain modules).
	Tognoli et al., 2007, Proceedings of the National Academy of Sciences of the United States of America	Pairs of subjects sat in front of each other while executing self-paced rhythmic finger movements during 1-min trials.	16	High-resolution spectral analysis of electrical brain activity before and during visually mediated social coordination revealed a marked depression in occipital alpha and rolandic mu rhythms during social interaction that was independent of whether behavior was coordinated or not. In contrast, a pair of oscillatory components (phi_1_ and phi_2_) located above right centro-parietal cortex distinguished effective from ineffective coordination: increase of phi_1_ favored independent behavior and increase of phi_2_ favored coordinated behavior.
	Yun et al., 2012, Scientific Reports	Participants were instructed to look at the other participant's finger while holding his own finger as stationary as possible.	20	Synchrony of both fingertip movement and neural activity between the two participants increased after cooperative interaction.
Shared attention	Dikker et al., 2017, Current Biology	Twelve high school students engaged in a semester during regular classroom activities such as group discussion.	12	Students' brain-to-brain group synchrony predicts classroom engagement and social dynamics.
	Jahng et al., 2017, NeuroImage	Prisoner's Dilemma Game.	56	The EEG hyperscanning identified temporal dynamics and inter-brain synchronization across the cortex, providing evidence for involvement of these regions in the processing of face-to-face cues to read each other's intent to cooperate. Most notably, the power of the alpha frequency band (8–13 Hz) in the right temporoparietal region immediately after seeing a round outcome significantly differed between face-to-face and face-blocked conditions and predicted whether an individual would adopt a “cooperation” or “defection” strategy. Moreover, inter-brain synchronies within this time and frequency range reflected the use of these strategies.
	Lachat et al., 2012, frontiers in Human Neuroscience	In socially driven instructions, the participants had to follow explicitly their partner's gaze, while in color-driven instructions, the objects to be looked at were designated at by their color so that no explicit gaze following was required.	29	Joint attention periods—as compared to the no-joint attention periods—were associated with a decrease of signal power between 11 and 13 Hz over a large set of left centro-parieto-occipital electrodes, encompassing the scalp regions where alpha and mu rhythms have been described. This 11–13 Hz signal power decrease was observed independently of the task instruction: it was similar when joint versus no-joint attention situations were socially driven and when they were color-driven.
	Leong et al., 2017, Proceedings of the National Academy of Sciences of the United States of America	In experiment 1, infants viewed videos of an adult who was singing nursery rhymes with direct gaze (looking forward), indirect gaze (head and eyes averted by 20°), or direct-oblique gaze (head averted but eyes orientated forward). In experiment 2, infants viewed the same adult in a live context, singing with direct or indirect gaze.	36	Across both experiments, the adult had a significant (Granger) causal influence on infants' neural activity, which was stronger during direct and direct-oblique gaze relative to indirect gaze. During live interactions, infants also influenced the adult more during direct than indirect gaze. Further, infants vocalized more frequently during live direct gaze, and individual infants who vocalized longer also elicited stronger synchronization from the adult.
	van den Heuvel et al., 2018, Clinical Neurophysiology	A lateral weight-shifting task.	24	For congruent visual feedback no significant differences in cortical activity between the two groups were present. For incongruent visual feedback, the Parkinson's disease group showed significantly higher beta modulation in primary motor cortex, and higher alpha modulation in primary visual cortex.
Interactive decision-making	Balconi and Vanutelli, 2016, frontiers in Psychology	Subjects were required to develop a strategy to obtain a better outcome than a competitor (in term of error rate, and response time, RT).	24	A decreased left alpha activity (increased brain response) for post-feedback compared to pre-feedback condition was observed.
	Hu et al., 2018, Biological Psychology	Prisoner's Dilemma Game.	30	There was a higher cooperation rate and larger theta/alpha-band inter-brain synchrony in condition human–human than in human-machine. In the condition human–human, there were larger centro-frontal theta band and centro-parietal alpha-band inter-brain synchrony in tasks set for high cooperation (higher cooperation index vs. lower cooperation index). Enhanced inter-brain synchrony covaried with increased cooperative choices observed between lower cooperation index and higher cooperation index. Furthermore, a subjective measure of perceived cooperativeness mediated the relationship between game context and inter-brain synchrony.
	Jahng et al., 2017, NeuroImage	Prisoner's Dilemma Game.	56	The EEG hyperscanning identified temporal dynamics and inter-brain synchronization across the cortex, providing evidence for involvement of these regions in the processing of face-to-face cues to read each other's intent to cooperate. Most notably, the power of the alpha frequency band (8–13 Hz) in the right temporoparietal region immediately after seeing a round outcome significantly differed between face-to-face and face-blocked conditions and predicted whether an individual would adopt a “cooperation” or “defection” strategy. Moreover, inter-brain synchronies within this time and frequency range reflected the use of these strategies.
	Kawasaki et al., 2013, Scientific Reports	Alternating speech tasks.	40	Speech rhythms were more likely to become synchronized in human–human tasks than human–machine tasks. Moreover, theta/alpha (6–12 Hz) amplitudes synchronized in the same temporal and lateral-parietal regions in each pair. Behavioral and inter-brain synchronizations were enhanced after human–machine tasks.
Affective communication	Dumas et al., 2010, PLoS ONE	Participants were engaged in spontaneous imitation of hand movements.	18	Symmetrical increase in PLV was found between the right parietal regions of the model (CP6, P8) and of the imitator (CP6, P4, P8) in the alpha-mu frequency band. The central region (FC1, Cz) of the model's brain and the parieto-occipital brain region (P8, PO2, PO10) of the imitator were synchronized in the beta frequency band. A wide frontal central area (F4, FC2, Czar, C4, CP6) of the model's brain was synchronized with the parietal area (CP2, PZ, P4, P8, PO2, PO10) of the imitator's brain for the gamma frequency band.
	Goldstein et al., 2018, Proceedings of the National Academy of Sciences of the United States of America	Romantic partners were assigned the roles of target (pain receiver) and observer (pain observer) under pain–no-pain and touch–no-touch conditions. The women were asked to rate their pain intensity 2 s before the end of each condition using the numerical pain scale. Concurrently, the male partners were instructed to rate their partners' level of pain. Both partners wrote the number on a small piece of paper not visible to the other member of the couple.	42	Hand-holding during pain administration increases brain-to-brain coupling in a network that mainly involves the central regions of the pain target and the right hemisphere of the pain observer. Moreover, brain-to-brain coupling in this network was found to correlate with analgesia magnitude and observer's empathic accuracy.
	Konvalinka et al., 2014, NeuroImage	A synchronized finger-tapping task.	18	The interactive condition was characterized by a stronger suppression of alpha and low-beta oscillations over motor and frontal areas in contrast to the non-interactive computer condition.
	Müller and Lindenberger, 2011, PLoS ONE	Participants were aligned in a predetermined position with the 11 singers facing the conductor and standing in two rows. They engaged in choir singing.	12	Phase synchronization both in respiration and heart rate variability increase significantly during singing relative to a rest condition. Phase synchronization is higher when singing in unison than when singing pieces with multiple voice parts. Directed coupling measures are consistent with the presence of causal effects of the conductor on the singers at high modulation frequencies. The different voices of the choir are reflected in network analyses of cardiac and respiratory activity based on graph theory.
	Wang et al., 2015, PLoS ONE	Participants watched emotional movies together, seated side-by-side. Participants were required to refrain from talking and making gross movements throughout the whole experiment.	78	The autonomic signals of co-present participants were idiosyncratically synchronized and that the degree of this synchronization was correlated with the convergence of their emotional responses.

*This table included empirical researches related to EEG-based hyperscanning*.

Firstly, the psychological significance of inter-brain synchrony is unclear, neither is the minimum inter-brain synchrony requirements. The inter-brain synchrony between brains has been found in most of these studies above-mentioned. There are two patterns of inter-brain synchrony, symmetric and asymmetric inter-brain coherence, among these EEG-based hyperscanning studies. Whether the asymmetric pattern of coupling can be interpreted as the differential roles and different psychological process of the partners during social interactions, the issue should be fully investigated in the future. A series of studies have demonstrated that the neural activities of two individuals are more synchronized when they did synchronized action (Lindenberger et al., 2009; Dumas et al., 2011; Yun et al., 2012; Kawasaki et al., 2013). Another series of studies have attributed the inter-brain synchrony to interpersonal mutual cooperation (Jahng et al., 2017; Hu et al., 2018). However, the inter-brain synchrony has been shown in some non-cooperation or non-interaction activities, for example, the inter-brain synchrony emerged among listeners who attended to the same speaker (Kuhlen et al., 2012), between subjects who participated in a task and the subjects who observed (Kawasaki et al., 2013; Ménoret et al., 2014), and between the participants who just sat facing each other (Goldstein et al., 2018). Participants in these studies performed the task in an independent way that interactions scarcely existed (Hu et al., 2018). Whether the inter-brain synchrony reflects the functional similarity in common task or a basic neural constitution of interpersonal interaction, the assumptions are needed to be verified by future researches. In addition, the mental constituent of these tasks involved in the psychological process of the coherence of inter-brain activities were complicated, including empathy, attention and closeness. Maybe it was the reason that the inter-brain synchrony got across different frequencies in different experiments. An interesting question arises about how to decompose the complicated mental constituent into basic psychological processes. Answers to that question will help us understand the synchronization effects better.

Secondly, EEG-based hyperscanning studies told little about how this inter-brain synchrony was generated (Hu et al., 2018). Most experimental paradigms over the past decades were correlated with long-term neural activities and these studies described the inter-brain activities as a result of ongoing social interactions. In most of these experimental paradigms, participant remained unaltered during the process of social interactions. However, social interactions are interrelated and interacted with each other and it is also a process of turn-taking interaction. It is important to set up well-designed experiments investigating neural transient dynamics related to the real reciprocal interactions. EEG-based hyperscanning studies still have a long way to go, with a view to the methodological challenges of studying brains interactive mechanisms of social interaction.

Thirdly, the inter-brain synchrony can be applied to groups with social cognitive impairment. For example, it is useful to compare social interactions between normal individuals and normal one as well as between normal individuals with abnormal one (e.g., individuals with social cognitive impairment). There may be difference in the inter-brain synchrony in these two different circumstances. Based on these research findings, researchers can ensure the corresponding brain regions to social cognitive impairment in order to provide efficient treatment. For example, impairment of reciprocal social interactions is regarded as a key sign of individuals with autism spectrum disorder (ASD; Qualls and Corbett, 2017). However, the etiology of ASD remains largely unknown. With the advantage of the ability to localize the epicenter of brain activation, fMRI-based hyperscanning can precisely detect the regions exhibiting inter-brain activation. In the future, we can combine EEG-based hyperscanning with fMRI-based hyperscanning to study social brain disorders and get a better knowledge of the social interaction mechanisms of patients with autism spectrum disorder or borderline personality disorder. In the end, we can localize the target and put forward the applicable therapies. For example, the behavioral synchrony between psychotherapists and patients can be improved to enhance their inter-brain synchrony in order to promote communications and attain good treatment effect.

Fourthly, future researches should pay more attention to the influencing factors of the inter-brain synchrony. According to Zhang and Liu (2018), four factors including the aim of communication, the object of communication, the form of communication and the content of communication, are influencing interpersonal neural synchronization. For example, Dumas et al. used a biophysical model to quantify the correlation between the anatomical and functional similarity of the two brains and inter-brain synchronizations. Therefore, future studies should employ the EEG-based hyperscanning technique to investigate more factors that may influence the inter-brain synchrony.

## Author contributions

SL and XZ: Conceive and writing frame design; DL, SL and XZ: Wrote the paper; DL, SL, XL, CZ, AL, CJ, YC, HW, and XZ: Revise the manuscript.

### Conflict of interest statement

The authors declare that the research was conducted in the absence of any commercial or financial relationships that could be construed as a potential conflict of interest.

## Footnotes

^1^The asymmetric brain-coupling in Dumas et al. (2010). meant that there was a neural coupling from the leader to the follower while there was no neural coupling from the follower to the leader. On the contrary, the symmetric brain-coupling means that mutual neural couplings.
