# Corrigendum: Interactive Brain Activity: Review and Progress on EEG-Based Hyperscanning in Social Interactions

**DOI:** 10.3389/fpsyg.2021.681900

**Published:** 2021-05-07

**Authors:** Difei Liu, Shen Liu, Xiaoming Liu, Chong Zhang, Aosika Li, Chenggong Jin, Yijun Chen, Hangwei Wang, Xiaochu Zhang

**Affiliations:** ^1^School of Humanities and Social Science, University of Science and Technology of China, Hefei, China; ^2^Department of Education, Hefei University, Hefei, China; ^3^School of Foreign Languages, Anhui Jianzhu University, Hefei, China; ^4^Department of Mechanical and Automation Engineering, The Chinese University of Hong Kong, Hong Kong, China; ^5^Department of Social and Behavioural Sciences, City University of Hong Kong, Hong Kong, China; ^6^CAS Key Laboratory of Brain Function and Disease, and School of Life Sciences, University of Science and Technology of China, Hefei, China; ^7^School of Chemistry and Materials Science, University of Science and Technology of China, Hefei, China; ^8^Hefei Medical Research Center on Alcohol Addiction, Anhui Mental Health Center, Hefei, China; ^9^Academy of Psychology and Behavior, Tianjin Normal University, Tianjin, China

**Keywords:** social interaction, EEG-based hyperscanning, inter-brain synchrony, phase coherence, inter-brain activities

In the original article, we neglected to include the funder Humanities and Social Science Project of Hefei University, 18RW12ZDA, to Difei Liu. The updated Acknowledgments section is shown below.

The authors apologize for this error and state that this does not change the scientific conclusions of the article in any way. The original article has been updated.

